# Metformin treatment of diverse Caenorhabditis species reveals the importance of genetic background in longevity and healthspan extension outcomes

**DOI:** 10.1111/acel.13093

**Published:** 2022-01-15

Ma, Y, Jun, GR, Chung, J, et al. CpG‐related SNPs in the MS4A region have a dose‐dependent effect on risk of late–onset Alzheimer disease. Aging Cell. 2019; 18:e12964. https://doi.org/10.1111/acel.12964

In the article “CpG‐related SNPs in the MS4A region have a dose‐dependent effect on risk of late–onset Alzheimer disease,” the authors would like to add co‐authors with corresponding affiliations due to the summary statistics in manuscript replication that they have contributed. Hence, the author byline and affiliation section should appear as:

Yiyi Ma ^1,2^, Gyungah R. Jun ^1,3,4^, Jaeyoon Chung ^1^, Xiaoling Zhang ^1,3^, Brian W. Kunkle ^5^, Adam C. Naj ^6,7^, Li‐San Wang^7^, Charles C. White ^8,9^, Benjamin Grenier‐Boley^10^, Philippe Amouyel^10^, Jean‐Charles Lambert^10^, The European Alzheimer’s Disease Initiative, Rebecca Sims^11,12^, Alfredo Ramirez^13,14^, Julie Williams^11,12^, Genetic and Environmental Risk in AD Consortium, Joshua C. Bis^15^, Cornelia M. van Duijn^16,17^, Sudha Seshadri^18,19^, Cohorts for Heart and Aging Research in Genomic Epidemiology Consortium, David A Bennett ^20^, Philip L. De Jager ^2,8,9^, Alzheimer’s Disease Genetics Consortium, Richard Mayeux ^21^, Jonathan L. Haines ^22^, Margaret A. Pericak‐Vance ^5^, Gerard D. Schellenberg ^7^, Lindsay A. Farrer ^1,3,4,23,24^, Kathryn L. Lunetta ^3^

1 Department of Medicine (Biomedical Genetics), Boston University School of Medicine, Boston, Massachusetts.

2 Center for Translational & Computational Neuroimmunology, Multiple Sclerosis Clinical Care and Research Center, Division of Neuroimmunology, Department of Neurology, Columbia University Medical Center, New York, New York.

3 Department of Biostatistics, Boston University School of Public Health, Boston, Massachusetts.

4 Department of Ophthalmology, Boston University School of Medicine, Boston, Massachusetts.

5 John P. Hussman Institute for Human Genomics, Miller School of Medicine, University of Miami, Miami, Florida.

6 Department of Biostatistics, Epidemiology, and Informatics, University of Pennsylvania Perelman School of Medicine, Philadelphia, Pennsylvania.

7 Penn Neurodegeneration Genomics Center, Department of Pathology and Laboratory Medicine, University of Pennsylvania, Philadelphia, Pennsylvania.

8 Program in Translational NeuroPsychiatric Genomics, Institute for the Neurosciences, Departments of Neurology and Psychiatry, Brigham and Women's, Boston, Massachusetts.

9 Program in Medical and Population Genetics, Broad Institute, Cambridge, Massachusetts.

10 Univ. Lille, Inserm, Centre Hosp. Univ Lille, Institut Pasteur de Lille, UMR1167 ‐ Labex DISTALZ ‐ RID‐AGE ‐ Risk factors and molecular determinants of aging‐related diseases, Epidemiology and Public Health Department, F‐59000 Lille, France

11 Division of Psychological Medicine and Clinical Neurosciences, MRC Centre for Neuropsychiatric Genetics and Genomics, Cardiff University, Cardiff, UK.

12 UK Dementia Research Institute at Cardiff, Cardiff University, Cardiff, UK.

13 Department for Neurodegenerative Diseases and Geriatric Psychiatry, University Hospital Bonn, Bonn, Germany.

14 Department of Psychiatry and Psychotherapy, University of Cologne, Cologne, Germany.

15 Cardiovascular Health Research Unit, Department of Medicine, University of Washington, Seattle, WA 98101 USA

16 Department of Epidemiology, Erasmus Medical Centre, Rotterdam, the Netherlands.

17 Nuffield Department of Population Health, Oxford University, Oxford, United Kingdom.

18 The Glenn Biggs Institute for Alzheimer’s and Neurodegenerative Diseases, UT Health San Antonio, San Antonio, TX 78229 USA

19 Boston University School of Medicine and the Framingham Heart Study, Boston, MA 02118 USA

20 Rush Alzheimer's Disease Center, Rush University Medical Center, Chicago, Illinois.

21 Department of Neurology and Sergievsky Center, Columbia University, New York, New York.

22 Department of Population & Quantitative Health Sciences, Case Western Reserve University, Cleveland Ohio.

23 Department of Neurology, Boston University School of Medicine, Boston, Massachusetts.

24 Department of Epidemiology, Boston University School of Public Health, Boston, Massachusetts.

On the other hand, the statement below should be added after the last sentence in the funding information section.

Infrastructure for the CHARGE Consortium is supported in part by the National Heart, Lung, and Blood Institute grant HL105756. This work is also supported by the grant AG049505 and AG054076.

Then, the sentence below should be the last entry in the author’s contribution section.

BG‐B, PA, J‐C L, AR, JW, JCB, CMvD, SS contributed summary statistics for replication.

Furthermore, the following sections should be included to “Appendix 1”:

**Members of the European Alzheimer’s Disease Initiative**

Céline Bellenguez^1,^, Vincent Chouraki^1^, Diana Zelenika^2^, Yoichiro Kamatani^3^, Marie‐Thérèse Bihoreau^3^, Florence Pasquier^4^, Olivier Hanon^5^, Dominique Campion^6^ **,** Claudine Berr^7^, Vincent Deramecourt^4^, Luc Letenneur^8^, Kristel Sleegers^9,10^, Nathalie Fiévet^1^, Lina Keller^12^, Pascale Barberger‐Gateau^8^, Carole Dufouil^8^, David Wallon^6^, Jordi Clarimon^13,14^, Alberti Lleo^13,14^, Paola Bossù^15^, Gianfranco Spalletta^15^, Sandro Sorbi^16,17^, Florentino Sanchez Garcia^18^, Maria Candida Deniz Naranjo^18^, Paolo Bosco^19^, Daniela Galimberti^20^, Michelangelo Mancuso^21^, Patrizia Mecocci^22^, Maria Del Zompo^23^, Alberto Pilotto^24^, Maria Bullido^25,26,27^, Francesco Panza^28^, Paolo Caffarra^29,30^, Benedetta Nacmias^16,17^, Lars Lannfelt^97^, Martin Ingelsson^31^, Victoria Alvarez^32^, Cristina Razquin^33^, Pau Pastor^33,34^, Ignacio Mateo^35^, Onofre Combarros^34^, Hilkka Soininen^36,37^, Laura Fratiglioni^38,39^, Karolien Bettens^9,10^, Alexis Brice^40,41^Didier Hannequin^6^, Karen Ritchie^7,42^, Mikko Hiltunen^36,37^, Jean‐François Dartigues^8,43^, Christophe Tzourio^8^, Caroline Graff^39,44^, Mark Lathrop2^2,3,45^, Christine Van Broeckhoven^9,10^.

^1^Univ. Lille, Inserm, Institut Pasteur de Lille, UMR1167 ‐ Labex DISTALZ ‐ RID‐AGE ‐ Risk factors and molecular determinants of aging‐related diseases, Epidemiology and Public Health Department, F‐59000 Lille, France; ^2^Centre National de Genotypage, Institut Genomique, Commissariat à l'énergie Atomique, Evry, France; ^3^Fondation Jean Dausset, CEPH, Paris, France; ^4^Univ. Lille, Inserm, Centre Hosp. Univ Lille, Lille, 59000, France; ^5^University Paris Descartes, Sorbonne Paris V; Broca Hospital, Geriatrics department; Paris, France; ^6^CNR‐MAJ; Inserm, U1079; Rouen University Hospital; 76031 France, Rouen, France ; ^7^Inserm, U1061; Faculty of Medicine; Hôpital La Colombière; Montpellier, France; ^8^Inserm, U897; Victor Segalen University; F‐33076, Bordeaux, France; ^9^Neurodegenerative Brain Diseases Group, Department of Molecular Genetics, VIB, Antwerp, Belgium; ^10^Department of Molecular Genetics, VIB, Laboratory of Neurogenetics, Institute Born‐Bunge, University of Antwerp, Antwerp, Belgium; ^12^Aging Reasearch Center, Dept Neurobiology, Care Sciences and Society, Karolinska Institutet and Stockholm University, Stockholm, Sweden; ^13^Neurology Department. IIB Sant Pau. Sant Pau Hospital. Universitat Autònoma de Barcelona, Barcelona, Spain; ^14^Center for Networker Biomedical Research in Neurodegenerative Diseases (CIBERNED), Barcelona, Spain; ^15^Clinical and Behavioral Neurology, Fondazione Santa Lucia, Roma, Italy; ^16^NEUROFARBA Department of Neuroscience, Psychology, Drug Research and Child Health, University of Florence, Florence, Italy; ^17^Centro di Ricerca, Trasferimento e Alta Formazione DENOTHE, University of Florence, Florence, Italy; ^18^Department of Immunology, Hospital Universitario Dr. Negrin, Las Palmas de Gran Canaria, Spain; ^19^IRCCS Associazione Oasi Maria SS, Troina, Italy; ^20^University of Milan, Fondazione Cà Granda, IRCCS Ospedale Policlinico, Milan, Italy; ^21^Neurological Clinic, University of Pisa, Italy; ^22^Section of Gerontology and Geriatrics, Department of Clinical and Experimental Medicine, University of Perugia, Perugia, Italy; ^23^Section of Neuroscience and Clinical Pharmacology, Department of Biomedical Sciences, University of Cagliari, Cagliari, Italy; ^24^Gerontology and Geriatrics Research Laboratory, I.R.C.C.S. Casa Sollievo della Sofferenza, San Giovanni Rotondo (FG), Italy; ^25^Centro de Biología Molecular Severo Ochoa (CSIC‐UAM); Madrid, Spain; ^26^Centro de Investigación Biomédica en Red sobre Enfermedades Neurodegenerativas (CIBERNED), Madrid, Spain; ^27^Instituto de Investigación Sanitaria “Hospital la Paz” (IdIPaz), Madrid, Spain; ^28^Departement of Geriatrics,Center for Aging Brain ,University of Bari, Bari, Italy; ^29^Department of Neuroscience‐University of Parma, Parma, Italy; ^30^Center for Cognitive Disorders AUSL, Parma, Italy; ^31^Dept. of Public Health/Geriatrics, Uppsala University, Uppsala, Sweden; ^32^Genetica molecular‐Huca‐Oviedo, Oviedo, Spain; ^33^Neurogenetics Laboratory, Division of Neurosciences, Center for Applied Medical Research, University of Navarra School of Medicine, Pamplona, Spain; ^34^CIBERNED, Centro de Investigación Biomédica en Red de Enfermedades Neurodegenerativas, Instituto de Salud Carlos III, Spain; ^35^Neurology Service and CIBERNED, "Marqués de Valdecilla" University Hospital (University of Cantabria and IFIMAV), Santander, Spain; ^36^Institute of Clinical Medicine ‐ Neurology, University of Eastern Finland, Kuopio, 70211 Finland; ^37^Department of Neurology, Kuopio University Hospital, FIN‐70211 Kuopio, Finland; ^38^Aging Reasearch Center, Dept Neurobiology, Care Sciences and Society, Karolinska Institutet and Stockholm University, Stockholm, Sweden; ^39^Dept Geriatric Medicine, Genetics Unit, Karolinska University Hospital Huddinge, S‐14186 Stockholm; ^40^INSERM UMR_S975‐CNRS UMR 7225, Université Pierre et Marie Curie, Centre de recherche de l'Institut du Cerveau et de la Moëlle épinière‐CRICM, Hôpital de la Salpêtrière, Paris France; ^41^AP‐HP, Hôpital de la Pitié‐Salpêtrière, Paris, France; ^42^Imperial College, London ; ^43^Centre de Mémoire de Ressources et de Recherche de Bordeaux, CHU de Bordeaux, Bordeaux, France; ^44^Karolinska Institutet, Dept Neurobiology, Care Sciences and Society, KIADRC, Novum floor 5, S14186 Stockholm, Sweden; ^45^McGill University and Génome Québec Innovation Centre, Montreal, Canada

**Members of the Genetic and Environmental Risk in AD Consortium**

Denise Harold^1^, Rebecca Sims^2^, Nicola Denning^2^, Charlene Thomas^2^, Amy Gerrish^3^, Melanie L Dunstan^4^, Paul Hollingworth^5^, Jade Chapman^6^, Alexandra Stretton^6^, Angharad Morgan^6^, Patrick G Kehoe^7^, Christopher Medway^8^, Jenny Lord^8^, James Turton^8^, Nigel M Hooper^9^, Emma Vardy^10^, Jason D Warren^11^, Jonathan M Schott^11^, James Uphill^12^, Natalie Ryan^13^, Martin Rossor^11,13^, Yoav Ben‐Shlomo^14^, Daniilidou Makrina^15^, Olymbia Gkatzima^15^, Michelle Lupton^16^, Maria Koutroumani^15^, Despoina Avramidou^15^, Antonia Germanou^15^, Frank Jessen^17‐19^, Steffi Riedel‐Heller^20^, Martin Dichgans^21,22^, Reiner Heun^18^, Heike Kölsch^18^, Britta Schürmann^18^, Christine Herold^23^, André Lacour^23^, Dmitriy Drichel^24^, Per Hoffmann^25‐27^, Johannes Kornhuber^28^, Wei Gu^29^, Thomas Feulner^30^, Hendrik van den Bussche^31^, Brian Lawlor^32,33^, Aoibhinn Lynch^32,33^, David Mann^34^, A David Smith^35^, Donald Warden^35^, Gordon Wilcock^36^, Isabella Heuser^37^, Jens Wiltfang^38‐40^, Lutz Frölich^41^, Michael Hüll^42^, Kevin Mayo^43^, Gill Livingston^44^, Nicholas J Bass^44^, Hugh Gurling^45^, Andrew McQuillin^44^, Rhian Gwilliam^46^, Panagiotis Deloukas^47^, Ammar Al‐Chalabi^48^, Christopher E Shaw^48,49^, Andrew B Singleton^50^, Rita Guerreiro^51^, Karl‐Heinz Jöckel^52^, Norman Klopp^53^, H‐Erich Wichmann^54‐56^, Dennis W Dickson^57^, Neill R Graff‐Radford^57^, Li Ma^57^, Gina Bisceglio^57^, Elizabeth Fisher^58^, Nick Warner^59^, Stuart Pickering‐Brown^60^, David Craig^61^, Peter Passmore^61^, Bernadette McGuinness^61^, Janet A Johnston^61^, Stephen Todd^61^, Seth Love^62^, David C Rubinsztein^63^, Peter St George‐Hyslop^64‐66^, Nick C Fox^67^, John S K Kauwe^68^, John Hardy^69^, John Collinge^70^, Minerva M Carrasquillo^71^, Amanda J Myers^72^, Alfredo Ramirez^73‐75^, Simon Lovestone^76^, Carlos Cruchaga^77,78^, Clive Holmes^79^, Karen Ritchie^80^, Wolfgang Maier^81,82^; Tim Becker^81,83^, Susanne Moebus^84^, Kevin Morgan^85^, Kristelle Brown^85^, Markus M Nöthen^86^, John Gallacher^87^, Anthony Bayer^87^, John F Powell^88^, Petroula Proitsi^88^, Carol Brayne^89^, Michael Gill^90,91^, Fiona Matthews^92^, Simon Mead^93^, Robert Clarke^94^, Alison M Goate^95^, Michael J Owen^2^, Michael C O'Donovan^2^, Lesley Jones^2^, Peter A Holmans^2^, Valentina Escott‐Price^2^, Julie Williams^96^.

^1^School of Biotechnology, Dublin City University, Dublin 9, Ireland; ^2^Division of Psychological Medicine and Clinial Neurosciences, MRC Centre for Neuropsychiatric Genetics and Genomics, Cardiff University, UK; ^3^West Midlands Regional Genetics Service, Birmingham Women's and Children's NHS Foundation Trust, Birmingham, UK; ^4^Kennedy Institute of Rheumatology (NDORMS), University of Oxford, UK; ^5^Department of Psychology and Counselling, Cardiff and Vale University Health board, UK; ^6^Division of Psychological Medicine and Clinical Neurosciences, MRC Centre for Neuropsychiatric Genetics & Genomics, Cardiff University, UK; ^7^Bristol Medical School, University of Bristol, Southmead Hospital, Bristol, UK; ^8^Institute of Genetics, Queens Medical Centre, University of Nottingham, Nottingham, UK; ^9^Division of Neuroscience and Experimental Psychology, School of Biological Sciences, Faculty of Biology, Medicine and Health, University of Manchester, Manchester Academic Health Science Centre, Manchester M13 9PT, UK; ^10^Institute for Ageing and Health, Newcastle University, Biomedical Research Building, Campus for Ageing and Vitality, Newcastle upon Tyne; ^11^Dementia Research Centre, Department of Neurodegenerative Disease, UCL Institute of Neurology, London, UK; ^12^Department of Neurodegenerative Disease, MRC Prion Unit at UCL, Institute of Prion Diseases, London, UK; ^13^MRC Prion Unit at UCL, Institute of Prion Diseases, London; ^14^Population Health Sciences, Bristol Medical School, University of Bristol; ^15^Aristotle University of Thessaloniki, Despere 3, Thessaloniki, 54621, Greece; ^16^King’s College London, Institute of Psychiatry, Department of Neuroscience, De Crespigny Park, Denmark Hill, London, UK; ^17^German Center for Neurodegenerative Diseases (DZNE), 53175, Bonn, Germany; ^18^Department of Psychiatry and Psychotherapy, University of Bonn, 53127, Bonn, Germany; ^19^Department of Psychiatry and Psychotherapy, University of Cologne, 50937 Cologne, Germany; ^20^Institute of Social Medicine, Occupational Health and Public Health, University of Leipzig, 04103 Leipzig, Germany; ^21^Institute for Stroke and Dementia Research, Klinikum der Universität München, Munich, Germany; ^22^German Center for Neurodegenerative Diseases (DZNE, Munich), Munich, 80336, Germany; ^23^Deutsches Zentrum für Neurodegenerative Erkrankungen (DZNE, Bonn), Bonn, Germany; ^24^Cologne Center for Genomics, University of Cologne, Cologne, Germany; ^25^Institute of Human Genetics, University of Bonn, Bonn, Germany; ^26^Department of Genomics, Life & Brain Center, University of Bonn, 53127, Bonn, Germany; ^27^Division of Medical Genetics, University Hospital and Department of Biomedicine, University of Basel, CH‐4058, Basel, Switzerland; ^28^Department of Psychiatry and Psychotherapy, University of Erlangen‐Nuremberg, Germany; ^29^Department of Psychiatry and Psychotherapy, University Hospital, Saarland, Germany; ^30^Dept. Of Psychiatry, University Hospital, Saarland; ^31^Institute of Primary Medical Care, University Medical Center Hamburg‐Eppendorf, Germany; ^32^Mercer's Institute for Research on Ageing, St James' Hospital, Dublin; ^33^James Hospital and Trinity College, Dublin, Ireland; ^34^Division of Neuroscience and Experimental Psychology, School of Biological Sciences, Faculty of Biology, Medicine and Health, University of Manchester, Manchester Academic Health Science Centre, Manchester M13 9PT, UK; ^35^OPTIMA, Oxford University, Dept of Pharmacology, Mansfield Rd, Oxford OX1 3QT; ^36^Formerly Director of The Oxford Project To Investigate Memory and Ageing, Nuffield Department of Clinical Neurosciences, University of Oxford, West Wing, John Radcliffe Hospital, Oxford. OX3 9DJ; ^37^Department of Psychiatry, Charité Berlin, Germany; ^38^Department of Psychiatry and Psychotherapy, University Medical Center Goettingen (UMG), Germany; ^39^German Center for Neurodegenerative Diseases (DZNE), 37075 Goettingen, Germany; ^40^iBiMED, Medical Sciences Department, University of Aveiro, Aveiro, Portugal; ^41^Central Institute of Mental Health, Medical Faculty Mannheim, University of Heidelberg, Germany; ^42^Department of Psychiatry, University of Frankfurt am Main, Frankfurt am Main, Germany; ^43^Departments of Psychiatry, Neurology and Genetics, Washington University School of Medicine, St Louis, MO 63110, US.; ^44^Division of Psychiatry, University College London, UK; ^45^Department of Mental Health Sciences, University College London, UK; ^46^LGC, Unit 1‐2 Trident Ind Est, Pindar Rd, Hoddesdon, Herts, EN11 0WZ; ^47^The Wellcome Trust Sanger Institute, Wellcome Trust Genome Campus, Hinxton, Cambridge, UK.; ^48^Kings College London, Institute of Psychiatry, Psychology and Neuroscience, UK; ^49^UK Dementia Research Institute at King's College London, UK; ^50^Molecular Genetics Section, Laboratory of Neurogenetics, National Institute on Aging, National Institutes of Health, Bethesda, MD 20892, USA; ^51^LaboratoryofNeurogenetics,NationalInstituteonAging,NationalInstitutesofHealth,Bethesda,MD,20892, USA; ^52^Institute for Medical Informatics, Biometry and Epidemiology, University Hospital of Essen, University Duisburg‐Essen, Hufelandstr; ^53^Institute of Epidemiology, Helmholtz Zentrum München, German Research Center for Environmental Health, Neuherberg, Germany; ^54^Helmholtz Center Munich, Institute of Epidemiology, Neuherberg; ^55^Ludwig‐Maximilians University Chair of Epidemiology, Munich, Germany; ^56^Joint Biobank Munich and KORA Biobank; ^57^Department of Neuroscience, Mayo Clinic, Jacksonville, Florida, 32224, USA; ^58^Department of Neurodegenerative Disease, UCL Institute of Neurology, London, UK; ^59^Somerset Partnership NHS Trust, Somerset, UK; ^60^Institute of Brain, Behaviour and Mental Health, Faculty of Human and Medical Sciences, University of Manchester, Manchester, UK; ^61^Ageing Group, Centre for Public Health, School of Medicine, Dentistry and Biomedical Sciences, Queen's University Belfast, UK; ^62^Bristol Medical School, University of Bristol, Southmead Hospital, Bristol, UK; ^63^Cambridge Institute for Medical Research and UK Dementia Research Institute, University of Cambridge, Cambridge, UK; ^64^Cambridge Institute for Medical Research, University of Cambridge, Cambridge, UK; ^65^Department of Clinical Neurosciences, University of Cambridge, Cambridge, UK; ^66^Tanz Centre for Research in Neurodegenerative Disease, University of Toronto, Toronto, Ontario, Canada; ^67^Dementia Research Centre, Department of Neurodegenerative Disease, UCL Institute of Neurology, London, UK; ^68^Department of Biology, Brigham Young University, Provo, Utah, USA; ^69^Department of Molecular Neuroscience, UCL, Institute of Neurology, London, UK; ^70^Department of Neurodegenerative Disease, MRC Prion Unit at UCL, Institute of Prion Diseases, London, UK; ^71^Department of Neuroscience, Mayo Clinic, Jacksonville, Florida, USA; ^72^Department of Psychiatry and Behavioral Sciences, Miller School of Medicine, University of Miami, Miami, Florida, USA; ^73^Department of Psychiatry and Psychotherapy, University Hospital Cologne, Cologne, Germany; ^74^Department for Neurodegenerative Diseases and Geriatric Psychiatry, University Hospital Bonn, Bonn, Germany; ^75^Institute of Human Genetics, University of Bonn, Bonn, Germany; ^76^Department of Psychiatry, University of Oxford, Oxford, UK; ^77^Department of Psychiatry, Washington University School of Medicine, St. Louis Missouri, USA; ^78^Hope Center Program on Protein Aggregation and Neurodegeneration, Washington University School of Medicine, St. Louis, Missouri, USA; ^79^Division of Clinical Neurosciences, School of Medicine, University of Southampton, Southampton, UK; ^80^Faculty of Medicine, Imperial College, St. Mary's Hospital, London, UK; ^81^German Center for Neurodegenerative Diseases (DZNE), Bonn, Germany; ^82^Department of Psychiatry and Psychotherapy, University of Bonn, Bonn, Germany; ^83^Institute for Medical Biometry, Informatics and Epidemiology, University of Bonn, Bonn, Germany; ^84^Institute for Medical Informatics, Biometry and Epidemiology, University Hospital of Essen, University Duisburg‐Essen, Hufelandstr, 55, D‐45147 Essen, Germany; ^85^Institute of Genetics, Queen's Medical Centre, University of Nottingham, Nottingham, UK; ^86^Institute of Human Genetics, Department of Genomics, Life and Brain Center, University of Bonn, Bonn, Germany; ^87^Institute of Primary Care and Public Health, Cardiff University, Neuadd Meirionnydd, University Hospital of Wales, Heath Park, Cardiff UK; ^88^Institute of Psychiatry, King's College London, Denmark Hill, London, UK; ^89^Institute of Public Health, University of Cambridge, Cambridge, UK; ^90^Mercer's Institute for Research on Ageing, St James' Hospital, Dublin; ^91^James Hospital and Trinity College, Dublin, Ireland; ^92^MRC Biostatistics Unit, Cambridge, UK; ^93^MRC Prion Unit, Department of Neurodegenerative Disease, University College London Institute of Neurology, London, UK; ^94^Oxford Healthy Aging Project (OHAP), Clinical Trial Service Unit, University of Oxford, Oxford, UK; ^95^Ronald M. Loeb Center for Alzheimer's Disease, Icahn School of Medicine, Mount Sinai, New York; ^96^UK Dementia Research Institute at Cardiff, Cardiff University, CF24 4HQ.

**Members of the Cohorts for Heart and Aging Research in Genomic Epidemiology Consortium**

Joshua C. Bis^1^, Sven J. van der Lee^2^, Johanna Jakobsdottir^3^, Sonia Moreno‐Grau^4,5^, Yuning Chen^6^, Shahzad Ahmad^7^, Merce Boada^4,5^, Annette L. Fitzpatrick^8,9^, Albert V. Smith^10,11^, Xueqiu Jian^12^, Seung‐Hoan Choi^4^, Hieab H. Adams^7^, Jennifer A. Brody^1^, Gudny Eiriksdottir^3^, Thor Aspelund^3,13^, Edith Hofer^14,25^, Chloe Sarnowski^6^, Charles C. White^16^, Dina Vojinovic^2^, L.Adrienne Cupples^6,17^, Jayanadra J. Himali^6,17,18^, Adela Orellana^4^, Melissa E. Garcia^19^, Honghuang Lin^20^, Hata Comic^21^, Gena Roschupkin^7^, Shuo Li^6^, Isabel Hernández^4,5^, Qiong Yang^6^, Alexa S. Beiser^6,17,18^, Lluis Tarraga^4,5^, Thomas H. Mosley^22^, WT Longstreth^9,23^, Jr, Fernando Rivadeneira^7,24,25^, Eric Boerwinkle^26,27^, Jerome I. Rotter^28^, Andre G. Uitterlinden^7,24,25^, Itziar de Rojas^4,5^, Inés Quintela^29^, Ángel Carracedo^29^, Luis M. Real^30^, Yasaman Saba^31^, Oscar L. Lopez^32‐34^, Myriam Fornage^35^, Najaf Amin^7^, Lenore J. Launer^19^, M. Arfan Ikram^7,36,37^, Helena Schmidt^14,31^, Reinhold Schmidt^14^, Anita L. DeStefano^6,18^, Vilmundur Gudnason^3,11^, Bruce M. Psaty^1,9,38,39^, Agustin Ruiz^4,5^, Cornelia M. van Duijn^7,40^, Sudha Seshadri^41,42^.

^1^Cardiovascular Health Research Unit, Department of Medicine, University of Washington, Seattle, WA 98101 USA; ^2^Department of Epidemiology, Erasmus MC University Medical Center, Rotterdam, 3015CN the Netherlands; ^3^Icelandic Heart Association, Kopavogur, Iceland; ^4^Research Center and Memory Clinic of Fundació ACE, Institut Català de Neurociències Aplicades‐Universitat Internacional de Catalunya, Barcelona, Spain; ^5^Centro de Investigación Biomédica en Red de Enfermedades Neurodegenerativas, Instituto de Salud Carlos III, Madrid, Spain; ^6^Department of Biostatistics, Boston University School of Public Health, Boston, MA, USA; ^7^Department of Epidemiology, Erasmus Medical Center, Rotterdam, the Netherlands; ^8^Department of Family Medicine, University of Washington, Seattle, WA, USA; ^9^Department of Epidemiology, University of Washington, Seattle, WA, USA; ^10^Department of Biostatistics, University of Michigan, Ann Arbor, MI, USA; ^11^Faculty of Medicine, University of Iceland, Reykjavik, Iceland; ^12^Brown Foundation Institute of Molecular Medicine, University of Texas Health Sciences Center at Houston, Houston, TX, USA; ^13^Centre for Public Health, University of Iceland, Reykjavik, Iceland; ^14^Clinical Division of Neurogeriatrics, Department of Neurology, Medical University Graz, Graz, Austria; ^15^Institute for Medical Informatics, Statistics and Documentation, Medical University of Graz, Graz, Austria; ^16^Program in Medical and Population Genetics, Broad Institute, Cambridge, MA, USA; ^17^Framingham Heart Study, Framingham, MA, USA; ^18^Department of Neurology, Boston University School of Medicine, Boston, MA, USA; ^19^Laboratory of Epidemiology and Population Sciences, National Institute on Aging, Bethesda, MD, USA; ^20^Section of Computational Biomedicine, Department of Medicine, Boston University School of Medicine, Boston, MA, USA; ^21^Brain Center Rudolf Magnus, Department of Neurology and Neurosurgery, University Medical Center Utrecht, Utrecht, The Netherlands; ^21^Departments of Medicine, Geriatrics, Gerontology and Neurology, University of Mississippi Medical Center, Jackson, MS, USA; ^23^Department of Neurology, University of Washington, Seattle, WA, USA;^24^Department of Internal Medicine, Erasmus University Medical Center, Rotterdam, the Netherlands; ^25^Netherlands Consortium on Health Aging and National Genomics Initiative, Leiden, the Netherlands; ^26^School of Public Health, Human Genetics Center, University of Texas Health Science Center at Houston, Houston, TX, USA; ^27^Human Genome Sequencing Center, Baylor College of Medicine, Houston, TX, USA; ^28^Institute for Translational Genomics and Population Sciences, Departments of Pediatrics and Medicine, Los Angeles BioMedical Research Institute at Harbor‐UCLA Medical Center, Torrance, CA, USA;^29^Grupo de Medicina Xenomica, Universidade de Santiago de Compostela, Centro Nacional de Genotipado, Centro de Investigación Biomédica en Red de Enfermedades Raras, Santiago de Compostela, Spain; ^30^Unidad Clínica de Enfermedades Infecciosas y Microbiología, Hospital Universitario de Valme, Sevilla, Spain; ^31^Gottfried Schatz Research Center for Cell Signaling, Metabolism and Aging, Division of Molecular Biology and Biochemistry, Medical University Graz, Graz, Austria; ^32^Department of Psychiatry, University of Pittsburgh, Pittsburgh, PA, USA; ^33^Alzheimer's Disease Research Center, University of Pittsburgh, Pittsburgh, PA, USA; ^34^Department of Neurology, University of Pittsburgh, Pittsburgh, PA, US;^35^Brown Foundation Institute of Molecular Medicine, University of Texas Health Sciences Center at Houston, Houston, TX, USA; ^36^Department of Neurology, Erasmus MC University Medical Center, Rotterdam, the Netherlands; ^37^Departments of Radiology, Erasmus MC University Medical Center, Rotterdam, the Netherlands; ^38^Department of Health Services, University of Washington, Seattle, WA, USA; ^39^Kaiser Permanente, Washington Health Research Institute, Seattle, WA, USA; ^40^Nuffield Department of Population Health, Oxford University, Oxford, United Kingdo; ^41^The Glenn Biggs Institute for Alzheimer’s and Neurodegenerative Diseases, UT Health San Antonio, San Antonio, TX 78229 USA; ^42^Boston University School of Medicine and the Framingham Heart Study, Boston, MA 02118 USA.

The authors would like to apologize for the inconvenience caused.

